# Foundation model for comprehensive transcriptional regulation analysis

**DOI:** 10.1093/nsr/nwae355

**Published:** 2024-10-14

**Authors:** Zhaowei Yu, Yong Zhang

**Affiliations:** State Key Laboratory of Cardiovascular Diseases and Medical Innovation Center, Institute for Regenerative Medicine, Department of Neurosurgery, Shanghai East Hospital, Shanghai Key Laboratory of Signaling and Disease Research, Frontier Science Center for Stem Cell Research, School of Life Sciences and Technology, Tongji University, China; State Key Laboratory of Cardiovascular Diseases and Medical Innovation Center, Institute for Regenerative Medicine, Department of Neurosurgery, Shanghai East Hospital, Shanghai Key Laboratory of Signaling and Disease Research, Frontier Science Center for Stem Cell Research, School of Life Sciences and Technology, Tongji University, China

Transcription regulation is a complex biological process that researchers have long sought to decode. With recent advancements in artificial intelligence (AI), foundation models, particularly those based on transformer architectures, are emerging as promising tools to address this challenge. These models are first pre-trained to capture intricate patterns within input genomic data, such as DNA sequences and epigenomic or transcription factor binding information, mainly through self-supervised learning. This foundational training allows them to then be fine-tuned for specific biological contexts, predicting and interpreting how genomic features influence transcription regulation. This perspective reviews the development and applications of foundation models in transcription regulation, with a focus on their training methods, architectures, and the biological insights they provide into gene regulation. We also discuss the potential of multi-modal models, which integrate diverse data types to enhance predictive power and interpretability. With ongoing progress in model design and data integration, AI holds the potential to transform our understanding of gene expression and advance transcription regulation research.

Transcription regulation plays a pivotal role in controlling the fundamental process of flowing genetic information from DNA to RNA, as outlined in the central dogma of molecular biology. Decoding the complex architecture of transcription regulation is a central goal in biology [1]. This involves exploring key aspects such as identifying crucial *cis*-regulatory elements that influence gene expression, examining interactions between *trans*-regulatory proteins and these elements, understanding the organization of various *trans*-regulatory proteins, and ultimately deciphering how these components work together to impact gene expression. The complexity of this system, with millions of *cis*-regulatory elements and thousands of *trans*-regulatory proteins, results in significant challenges for elucidation. Furthermore, the limitations of high-throughput sequencing data, including its sparsity and heterogeneity, impede the development of universally accurate models for transcription regulation.

Artificial intelligence (AI) has revolutionized many aspects of human society, with life sciences being one of the most profoundly impacted fields. Recent advancements in large language models have achieved significant successes across natural language processing, computer vision and multi-modal applications [2–4]. These foundation models leverage transfer learning to effectively apply knowledge from pre-training on large-scale corpora to specialized downstream applications through fine-tuning [5]. This method enhances model performance and significantly reduces the time and resources required for model development. The advancements in foundation models and transfer learning have enabled broader applications of AI in life sciences, particularly in areas with biological sequences analogous to languages, such as protein sequences, DNA sequences and RNA sequences.

In this perspective, we explore the applications of advanced AI techniques with regard to modeling and understanding transcription regulation (Fig. 1). In the first part, we discuss the recent advancements in foundation models that enhance our comprehension of transcription regulation. This involves leveraging pre-trained large language models and transfer learning to deepen our insight into the complex biological process. In the second part, we outline our expectations for developing new multi-modality foundation models, which are expected to more accurately mirror real *in vivo* transcription regulation scenarios by integrating multi-modal information related to transcription regulation. Such integration is anticipated to enable more precise predictions and provide profound insights into the mechanisms of transcription regulation.

**Figure 1.**
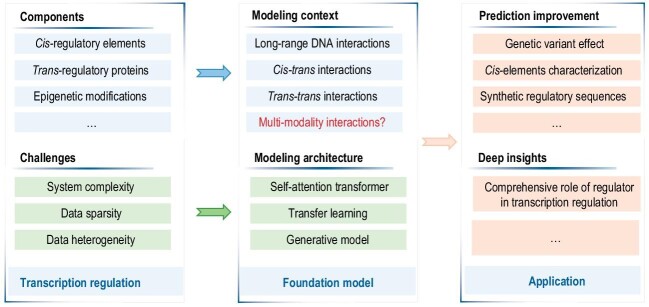
Recent advancements in AI for transcription regulation. Left, key components in transcription regulation and the challenges associated with understanding transcription regulation in cells. Center, contexts modeled by recent foundational models for transcription regulation, and model architectures highlighting their structures and learning strategies designed to address the challenges. The context, which the expected multi-modality foundation model would model, is labeled in red. Right, applications of foundational models, including improvements in prediction accuracy for various downstream tasks and comprehensive insights into the roles of *trans*-regulatory proteins.

## FOUNDATION MODELS OF TRANSCRIPTION REGULATION

Foundation models, especially large language models characterized by their extensive weight parameters, are highly effective at representing complex systems. These models are typically built on transformer architectures that include self-attention layers. This structure enables each element of an input sequence to be attended to in the context of all other elements, enhancing the model's ability to capture intricate relationships within the data. The robust representational capabilities of large language models are particularly advantageous for modeling complex transcription regulation *in vivo*. Moreover, the ability of foundational models to utilize transfer learning extends their usefulness across a diverse range of downstream tasks, especially in settings with sparse and heterogeneous data. This adaptability, combined with their generalization capabilities, has gained significant attention within the scientific community, highlighting their potential to accelerate advancements in transcription regulation.

A DNA sequence is composed of just four types of nucleotide bases, yet combinations of nucleotide sequences from adjacent genomic regions create a diverse array of *cis*-regulatory elements. Traditionally, bioinformatics has focused on decoding the ‘grammar’ of DNA by examining arrangements of short, contiguous nucleotide sequences known as *k*-mers. AI models like DeepBind [6], DeepSEA [7] and Basset [8] have employed convolutional neural networks to predict DNA/RNA-binding protein specificities and chromatin accessibility based on these *k*-mer patterns. However, this approach has not been sufficient to fully capture the complexity of DNA. In recent years, advanced deep-learning models, specifically large language models, have been employed to better understand DNA sequence organization. These models utilize self-attention mechanisms capable of processing long sequences, allowing them to discern complex relationships among nucleotide sequences over larger genomic windows. For instance, DeepMind's Enformer can model interactions within DNA sequences up to 100 kilobases away [9]. Similarly, DNABERT, a bidirectional encoder representations from transformers (BERT)-like transformer model, effectively captures interactions involving hundreds of *k*-mers [10]. These models have been pre-trained on large-scale data sets, enabling them to learn universal representations. These representations can enhance predictive capabilities and provide deeper insights into downstream tasks in settings where data or ground truths are limited. For example, Enformer has shown significant improvements in predicting the functional effects of DNA sequences, such as the impact of genetic variants on gene expression quantitative trait loci (eQTL) and in massively parallel reporter assays (MPRAs) [9]. Moreover, its attention mechanisms also help identify potential enhancer-gene pairs. Similarly, DNABERT has achieved success in predicting *cis*-regulatory elements like promoters, splice sites and transcription factor binding sites, demonstrating its effectiveness in genome-wide regulatory element characterization [10].

While both DNABERT and Enformer use a transformer for DNA sequence modeling, they differ in their pre-training strategies and the genomic regions they focus on. DNABERT employs a masked language modeling approach similar to BERT [2], learning contextual relationships within relatively narrow genomic regions during pre-training. This method makes the model well-suited for tasks that require precise DNA sequence information and enhances its ability to generalize effectively [10]. However, this focus limits the model's capacity to capture more complex organizational patterns within the genome. In contrast, Enformer utilizes a supervised learning approach to understand contextual relationships across broader genomic regions involved in regulating gene expression and epigenetic landscapes [9]. This enables Enformer to capture intricate regulatory relationships, such as long-range interactions between enhancers and promoters. Despite these advantages, the high computational resources required by Enformer and its specific pre-training tasks limit its direct applicability to a wide range of downstream tasks.

In cellular biology, *trans*-regulatory proteins play a critical role in transcription regulation. Yet, modeling their impact on transcription remains challenging due to the sparsity and heterogeneity of genome-wide profiles for these proteins, which vary for thousands of *trans*-regulatory proteins in many cell types and conditions. This significant lack of profiles impedes the accurate representation of these *cis*-*trans* and *trans*-*trans* interactions. Recent progress in foundation models has addressed these challenges by jointly modeling DNA sequences and the binding events of *trans*-regulatory proteins. For instance, cis-regulatory element auto translator (CREaTor) [11] is a foundation model designed to predict gene expression by integrating genomic sequences with chromatin accessibility and various epigenetic profiles. Furthermore, a pretrained transformer model for epigenomics, EpiGePT, [12] has pushed these boundaries further by including a module that characterizes the state of several hundred transcription factors, encompassing both their expression profiles and binding affinities in specific contexts. EpiGePT is adept at learning cell-type-specific chromatin contacts and identifying genetic variants associated with human diseases. However, despite incorporating a larger set of transcription factors than CREaTor, EpiGePT relies on the input of DNA-binding motifs for these factors. These motifs may not always reflect their true binding profiles, potentially limiting the model's ability to fully capture transcription factor interactions.

Recently, we developed ChromBERT (https://github.com/TongjiZhanglab/ChromBERT), which models the co-binding pattern of thousands of *trans*-regulatory proteins directly by pooling chromatin immunoprecipitation followed by sequencing (ChIP-seq) profiles from different cell types, which allows ChromBERT to learn a universal grammar for their context-specific combinatorial interactions. These advanced models significantly improve our understanding of the interactions within and between *trans*-regulatory proteins and DNA sequences, providing deep insights into the roles of *trans*-regulatory proteins in transcription regulation. For instance, ChromBERT facilitates the identification of critical regulators that influence cellular heterogeneity or cellular reprogramming by interpreting fine-tuned regulator embeddings, some of which are hard to infer in traditional analysis through differential expression or differential chromatin accessibility for a single regulator.

The success of advanced models, such as GPT-4, DALLE-3 and MidJourney, has heightened interest in using generative AI within synthetic biology [3]. This innovative technology allows researchers to explore and generate biological sequences that do not naturally occur, opening up new possibilities for therapeutic applications. In the field of transcription regulation, one of the successful applications of generative AI involves the generation of cell-type-specific enhancers from random sequences. Two previous studies have demonstrated the capability of deep-learning models to learn the *cis*-regulatory code associated with chromatin accessibility profiles, enabling the generation of enhancers that can regulate gene expression in a cell-type-specific manner [13,14]. Another notable application is DNA-Diffusion [15], a conditional diffusion model designed for generating DNA sequences specific to the given condition. Trained on diverse *cis*-regulatory elements across different conditions, DNA-Diffusion can synthesize DNA sequences tailored to specific regulatory needs.

## TOWARDS BUILDING MULTI-MODALITY FOUNDATION MODELS FOR TRANSCRIPTION REGULATION

Multi-modal integration has become a central focus in AI and has experienced rapid growth over the past few years. This approach combines diverse data types, such as text, images and audio, into unified representations [4]. While several foundation models have been developed for computational modeling of transcription regulation, multi-modal foundation models are expected to drive significant advancements in this field. Transcription regulation is a complex system influenced by various factors beyond *cis*-regulatory elements and *trans*-regulatory proteins. These include non-coding RNAs, chromatin structure, external signals and environmental factors. A multi-modality model that integrates a wide array of transcription regulatory effectors can more accurately reflect the complexity of transcription regulation. Such comprehensive models are expected to enhance the representation and predictive capabilities of foundation models, leading to more precise and insightful outcomes in the study of gene expression regulation. Additionally, they improve model interpretability, facilitating a better understanding of the interactions between different modalities. Finally, multi-modality foundation models enable AI-driven synthesis of a comprehensive transcription regulation system rather than focusing solely on *cis*-regulatory elements.

Establishing a multi-modality foundation model for transcription regulation remains a significant challenge for future studies. The primary difficulties include handling heterogeneous data sources and addressing data alignment issues. Building a multi-modality foundation model requires harmonizing data from diverse sources such as genomics sequences, transcriptomic profiles, transcription factor binding profiles, chromatin structures, RNA binding profiles and epigenetic modifications. To achieve this, the first step is to standardize these data sets, ensuring consistency and compatibility across modalities. Standardization methods generally fall into two categories: discretizing continuous values from high-throughput sequencing data and tokenizing sequences (such as DNA, RNA or protein sequences) through approaches like one-hot encoding, *k*-mer encoding or byte-pair encoding. These techniques must be carefully tailored to the specific characteristics of each data source. When it comes to data alignment, most modalities can be mapped to genomic coordinates. However, transcriptomic profiles pose a unique challenge, as gene expression is influenced by more than just the genomic regions where transcription starts. One approach to address this is to construct regulatory relationships using adjacent regions or long-range chromatin contacts, potentially through graph-based models. For example, graph-linked unified embedding (GLUE) has successfully applied this method to integrate single-cell multi-modality data [16]. Another major challenge is the completeness of the data. Incomplete data sets can lead to insufficient encoding and representation of transcriptional regulation. This limitation highlights the need for more comprehensive experimental data generation and collection from various modalities and the development of robust data quality control, processing and interpolation methods. Synthetic data generation, commonly used in computer vision and natural language processing, may offer a solution. For instance, ChromBERT can generate cistromes for ∼1000 transcription factors through prompt-enhanced fine-tuning, providing a valuable resource for developing multi-modality models.

In developing multi-modality foundation models for transcription regulation, the model architecture and learning strategy should also be specifically designed. Integrating multi-modal data requires careful consideration in model architecture for where to incorporate different modalities, either before or after feature embedding, and how to combine them through concatenation, addition or multiplication. The choice of architecture for modeling their crosstalk is also pivotal, with common options including multilayer perceptrons, convolutional neural networks and transformer blocks. Each choice impacts the model's ability to learn fundamental knowledge about how these transcription effectors jointly regulate gene expression. The model's generalization and flexibility are also important, as they are essential for applying the model to downstream tasks with specific condition settings. Transcription regulation typically occurs genome-wide, involving millions of genomic intervals. This scale necessitates substantial computational resources for both training and inference. To manage these demands effectively, learning strategies such as distributed computing, optimization of memory usage and acceleration of computation are critical. The learning strategies employed in these models are equally important. Self-supervised learning has become popular in the field due to the often-unavailable ground truth in cross-modal interactions. However, supervised learning also plays a crucial role by establishing a direct model from transcription regulatory effects to gene expression outcomes. Furthermore, the contributions of different modalities within the model can be strategically weighted based on prior biological knowledge.

After pre-training foundation models, a key challenge is applying them effectively to advance the field. Two crucial components of this process are fine-tuning for specific downstream tasks and interpreting the model's biological insights. Both are essential to fully realizing the potential of foundation models in transcription regulation research.

The success of fine-tuning depends on the size and relevance of the task-specific data set. In biology, data sets are often small, and their relationship to pre-training tasks can vary. When downstream tasks closely align with pre-training, fewer model parameters need adjustment. However, when the tasks differ significantly, more extensive retraining is required. Given that downstream tasks in life sciences are often data limited, efficient fine-tuning is essential to avoid overfitting and enhance the model's performance in few-shot scenarios. Another risk is ‘hallucination’, where inappropriate downstream tasks or inefficient fine-tuning strategies can cause the model to generate inaccurate predictions. Foundation models are pre-trained on large data sets that may contain both correct and incorrect information, leading to high generalization across domains. This can result in failures in highly specific or technical areas, where there is a lack of accurate ground truth in the pre-training data. Interpreting foundation models also poses a challenge, as deep learning models are often seen as ‘black boxes’ due to their complexity and vast number of parameters. In biological research, however, understanding why a model makes certain decisions and what its learned parameters and embeddings represent is crucial. Techniques such as feature importance analysis, including in silico perturbations, can help identify which input features contribute most to the model's predictions. In transformer-based architectures, attention scores highlight which parts of the input the model focuses on, providing insight into how different input features interact. Additionally, learned embeddings can offer valuable insight into the specific roles of various input elements. However, these interpretability analyses depend on the model's ability to accurately represent true biological processes. This requires careful consideration of the model architecture and may sacrifice the model performance.
